# Thrombocytopenic purpura on an organic farm with pen mating: a case report on the re-emergence of an old disease

**DOI:** 10.1186/s40813-020-00157-z

**Published:** 2020-08-04

**Authors:** Sara Joller, Irene M. Häfliger, Cord Drögemüller, Olivia K. Richard, Alexander Grahofer

**Affiliations:** 1 Clinic for Swine, Department of Clinical Veterinary Science, Vetsuisse Faculty, University of Bern, 3012 Bern, Switzerland; 2 Institute of Genetics, Vetsuisse Faculty, University of Bern, 3012 Bern, Switzerland; 3 Department of Infectious Diseases and Pathobiology, Institute of Animal Pathology, Vetsuisse Faculty, University of Bern, 3012 Bern, Switzerland

**Keywords:** Pig, Haemorrhagic disease, Genetic testing, Disease awareness

## Abstract

**Background:**

Thrombocytopenia is an immune-mediated disease, which affects suckling piglets. Piglets are pale and inactive, show multiple hemorrhages and often die within days. Pathological examination reveals severe haemorrhages and oedema in several organs. Severe thrombocytopenia and elongated bleeding time characterize the disease haematologically.

The sow produces antibodies against the thrombocyte antigens of the boar, which are present in the blood of the piglets. These isoimmune antibodies attack the platelets and megakaryocytes of the piglets, causing thrombocytopenia in succeeding matings of the same boar and sow. There is no known therapy against this condition. In the last few decades, the disease has become rare due to the increase of artificial insemination.

**Case presentation:**

On an organic breeding farm in Switzerland with a high percentage of natural pen matings, piglets of three litters showed haemorrhages on the skin, prolonged bleeding time and were generally in a reduced general state. A pathological examination revealed multifocal haemorrhages in the stomach, kidneys, dermis, mesenterium and spinal cord. Haematology showed a massive thrombocytopenia and regenerative anaemia. Due to these findings the diagnosis of thrombocytopenic purpura was established.

To avoid further matings of the same boar and sow and thus more affected piglets, out of three possible boars the responsible sire had to be determined. This was achieved through array genotyping and subsequent computation of identity by descent and calculation of Mendelian errors for parentage verification. Thereby the responsible boar was identified and as a consequence removed from the farm. Further preventive measures, that had been established, included the recording of all matings and regular exchange of boars.

**Conclusion:**

The decreased number of natural matings with the surge of artificial insemination has probably reduced the number of cases of thrombocytopenic purpura and thus the disease awareness of farmers and veterinarians. However, as consumers wish for better animal welfare and higher ecological standards we may see a rise in natural matings and thus a return of the disease. In case of affected litters, genetic testing was proven a valid method for investigation and prevention of more cases and may be used more in the future.

## Background

Thrombocytopenic purpura in suckling piglets is an immune-mediated disease that has been known in veterinary medicine for about 50 years [1–3]. The disease is characterized by massive haemorrhages in suckling piglets, caused by a thrombocytopenia. Piglets are pale and inactive and often die within 2 to 15 days [1–3]. Petechial and ecchymosal bleedings are usually first seen in the skin of the ventral abdomen and on ears [3–5]. The strongest piglets of the litter are often affected more severely [3]. Symptoms appear at 3 days of age and abate after a few days. At around 14 days of age the clinical symptoms reappear [3]. Similar case history is also described in Göttingen minipigs, but onset of symptoms occur at an older age [6].

Pathological examination reveals severe haemorrhages and oedema in several organs such as lung, heart, kidneys, subcutis, joints, mucosa of gastrointestinal and respiratory tract, skeletal muscles, brain and meninges [2–4, 7]. The heavier bleedings are found in more mobile organs, such as heart, diaphragm and skeletal muscles [3]. In addition, lymph nodes are found to be haemorrhagic and enlarged [1–3, 7]. Histologic findings include haemorrhages in several tissues and a depletion of megakaryocytes in bone marrow and spleen [2, 3, 8].

Haematological findings show a severe thrombocytopenia in affected piglets [1, 3, 8, 9], elongated bleeding time [1, 4, 8] but normal whole blood coagulation, prothrombin and activated partial thromboplastin times [1, 4, 10].

Thrombocytopenia develops due to isoimmune antibodies, which attack the platelets and megakaryocytes of the piglets [1–4]. The sow produces antibodies against the thrombocyte antigens of the boar, which are present in the blood of the piglets [3, 4]. Therefore, symptoms usually develop only in litters from the second mating onwards with the same boar [3] and can get worse in the following litters [7]. Isoimmunisation occurs more often in breedings between Landrace and Large White [3, 4]. The piglets ingest the isoimmune antibodies with the colostrum [3]. These maternal antibodies agglutinate the thrombocytes of the piglets, which causes symptoms to appear at about 3 days of age. The antibodies also have a cytotoxic effect on megakaryocytes, which are the precursor cells of the thrombocytes [2, 3, 8]. The second often more severe appearance of symptoms at age of 2 weeks is due to the destruction of platelets and megakaryocytes and the following failure to produce new platelets (biphasic thrombocytopenia) [3, 4]. Once a litter is affected of thrombocytopenic purpura, there are no therapeutical options. Newborn piglets of matings that are known to cause thrombocytopenic purpura should be separated from their mother before consumption of colostrum and fostered by another sow [8]. Subsequent matings of the sow and boar of the affected litter should be avoided. However, due to cross-fostering or insufficient documentation of breeding, identification of parents might be impossible [10]. The disease has become rare since the emergence of artificial insemination, which decreases the possibility of two identical matings. However, with the development of special agricultural programmes that place great emphasis on environmentally friendly and animal welfare-oriented pig farming, the proportion of natural matings has risen again [11, 12]. Emerging diseases such as African swine fever may cause a similar clinical picture and need to be differentiated from afflictions that are more harmless. Other differential diagnosis include Classical Swine fever, septicaemia and warfarin poisoning. At the same time disease awareness for thrombocytopenic purpura has decreased over time. Emerging cases may find practitioners unaware of this cause and best management of the disease. Such was the case in this report, where we discuss the case of three litters with thrombocytopenic purpura in an organic farm. The identification of the responsible sire was done by using current techniques for parentage verification.

## Case presentation

On a Swiss breeding farm with 70 sows (Landrace) and three boars (Large White), piglets of three litters showed haemorrhages in the skin. The farm worked according to the guidelines of the Swiss organic program “Bio Suisse” [13]. A 1 week batch farrowing system was conducted in the herd. Three boars performed 95% of all pen-matings, while artificial insemination was conducted in the rest of the sows. After weaning, the sows were transferred to the insemination unit and were split into groups of three to four sows. After 4 days, pen-mating was conducted with one boar per pen. The boars were rotating between the different pens daily. As common in pen-mating, an unsupervised mating process took place and therefore, the sire of the next litter was unknown. Sows and boars stayed together until the first pregnancy control after 3 weeks. During the gestation period the sows were group housed in a pen with deep straw bedding and a concrete outdoor area with a muddy pool filled with water. One week prior to farrowing the sows were moved into a free farrowing pen. The sows were fed a commercial feed for gestating sows during pregnancy once a day and a feed for lactating sows during lactation twice a day.

The first of the three affected litters was observed in the beginning of 2017, when two piglets died of prolonged bleeding after intramuscular injection of iron dextran 3 days after birth. Three other siblings of the remaining 12 piglets were observed to have haemorrhages of the skin and swollen joints and died within 3 days. The rest of the litter survived and no prolonged bleeding was observed during castration 2 weeks after birth. No further diagnostics were performed in this litter.

**Fig. 1:**
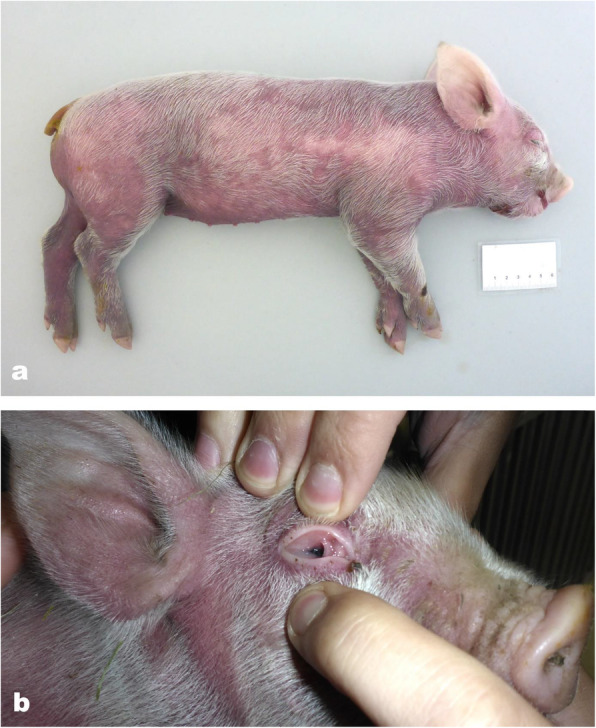
A piglet with thrombocytopenic purpura of the second affected litter. **a** Disseminated haemorrhages of a dark red to violet colour could be observed all over the skin. **b** The ocular mucous membranes of the affected piglets were pale

**Fig. 2:**
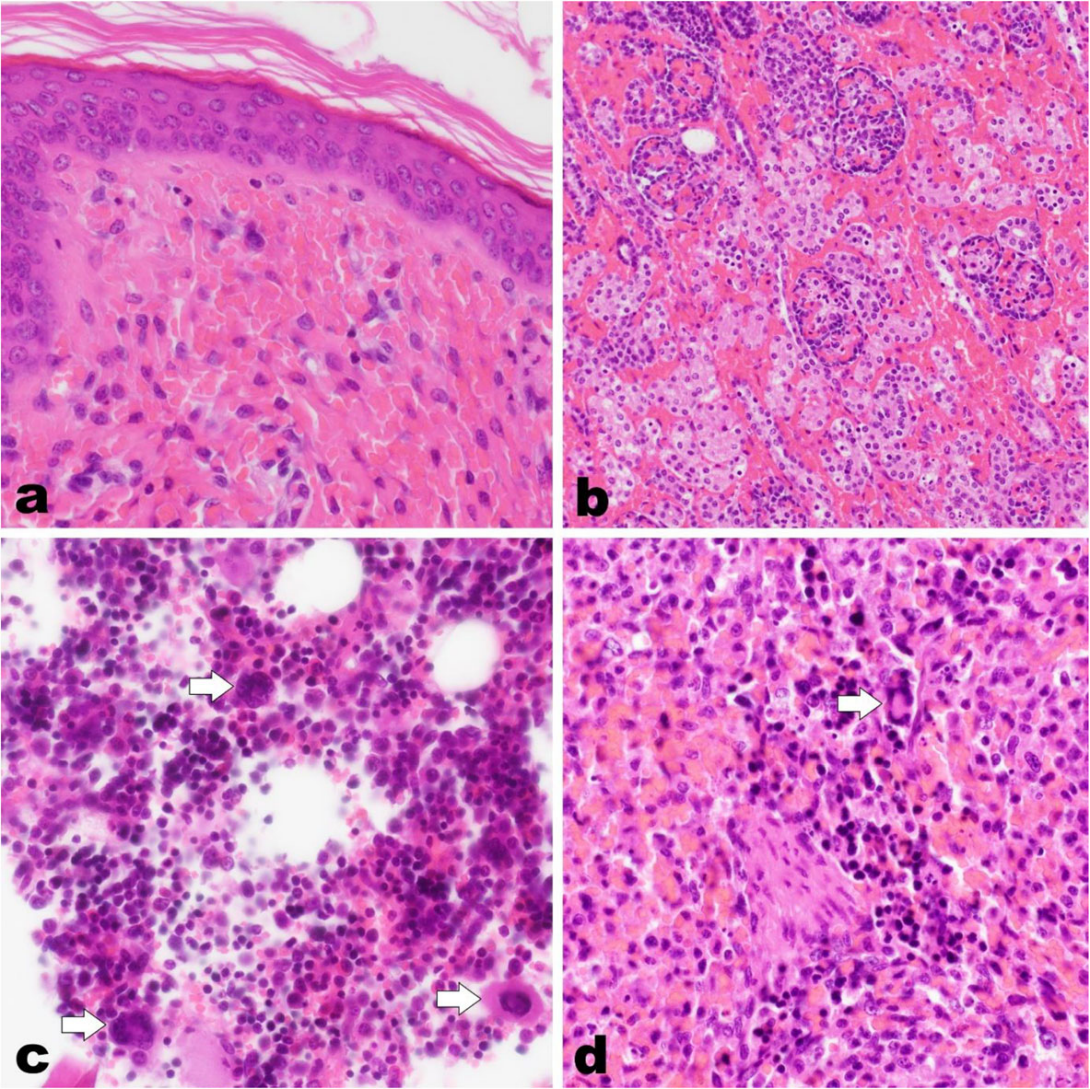
Histology of dermis, renal cortex, bone marrow and spleen of an affected piglet. Severe multifocal haemorrhages in the dermis (**a**) and the renal cortex (**b**) with a high number of extravasated erythrocytes. Hyperplastic bone marrow (**c**) with an overall increased cell number and high megakaryocyte count (arrows). Extramedullar haematopoiesis in the spleen (**d**) characterized by megakaryocytes (arrow) and haematopoietic progenitor cells. Haematoxylin and eosin stain, 40 x (**a**, **c**, **d**) and 20 x magnification (**b**)

Another litter showed similar symptoms 9 months later. The farmer observed disseminated haemorrhages and prolonged bleeding time after injection and only four of 14 piglets survived. At the Swine clinic in Bern, Switzerland, three dead and one live piglets were presented at 6 days of age with severe symptoms. The live piglet was apathetic. The skin of all four piglets was multifocal to coalescing of dark red, with the ventral abdomen and hind legs more severely affected (Fig. 1). The mucous membranes were pale. Blood was collected from the live piglet and it was euthanized. Necropsy of all four piglets revealed severe multifocal haemorrhages in the mucosa of the stomach as well as in the renal cortex. The bone marrow of the humerus was diffusely dark red. In histology, haemorrhages were present in the dermis, the renal medulla and cortex, the mesenterium and the spinal cord (Fig. 2). The bone marrow was diffusely hyperplastic with an overall increased cell number, especially megakaryocyte count was high. The liver and spleen were diffusely congested and multifocal extramedullar haematopoiesis was present in the spleen.

Blood was taken and analysed from the live piglet. A massive thrombocytopenia and regenerative anaemia were revealed through haematology (Table 1). Prothrombin time was slightly elongated. Potassium, urea and lactate dehydrogenase (LDH) concentrations were slightly elevated, while the total protein count was marginally too low due to a loss of albumin.

**Table 1:** Haemotology and clinical chemistry of one affected piglet

Haematology	Value	Reference value	Unit
**Haematocrit** [14]	**0.16**	22.0–31.0	l/l
**Erythrocytes** [14]	**2.59**	3.4–4.7	10^12^/l
**Haemoglobin** [14]	**44**	64–94	g/l
**MCV** [14]	62.1	60–74	fl
**MCH** [14]	17.1	17–23	pg
**MCHC** [14]	276	264–309	g/l
**RDW** [14]	21.7	5–54	%
**Thrombocytes** [15]	**89**	171.8–833.2	10^9^/l
**MPV** [15]	**30.6**	7.4–16.5	fl
**Leukocytes** [16]	14.02	10–22	10^9^/l
**Normoblasts** [16]	**0.77**	0	10^9^/l
**Banded neutrophils** [16]	1.05	0–1.5	10^9^/l
**Segmented neutrophils** [16]	7.99	1–8.2	10^9^/l
**Lymphocytes** [16]	**4.00**	6.0–16.0	10^9^/l
**Monocytes** [16]	0.77	0–1	10^9^/l
**Eosinophils** [16]	0.00	0–1.3	10^9^/l
**Basophils** [16]	0.00	0–0.05	10^9^/l
**Blasts (Zentrallabor, Vetsuisse Bern)**	**0.21**	0	10^9^/l
**Prothrombin time (PT)** [17]	**18.1**	15.2–17.4	sec
**Partial Thromboplastin Time (PTT)** [17]	**12.3**	15.5–20.1	sec
**Sodium** [15]	146	94–150	mmol/l
**Potassium** [15]	**7.54**	3.6–7.2	mmol/l
**Chloride** [15]	103	64–106	mmol/l
**Calcium** [15]	1.98	1.70–3.2	mmol/l
**Phosphorous** [15]	3.95	2.3–3.9	mmol/l
**Magnesium** [15]	1.47	0.9–1.5	mmol/l
**Serum iron** [15]	5.6	2–8.5	mmol/l
**Total protein** [15]	**29.6**	31.0–61.0	g/l
**Albumin** [15]	**16.8**	23–46	g/l
**Urea** [15]	**33.52**	0.9–4.9	mmol/l
**Creatinine** [15]	136	36.0–141.0	μmol/l
**Bilirubin** [15]	5.5	1–22	μmol/l
**ALAT** [16]	24	0–68	IU
**AP** [15]	1140	160–2119	IU
**ASAT** [15]	52	3–130	IU
**CK** [15]	472	111–4918.0	IU
**GLDH** [15]	3	0–65	IU
**LDH (Zentrallabor, Vetsuisse Bern)**	**2178**	909–2172	IU
**SDH** [16]	0	0–1	IU

Values not within the physiological bond are marked in bold

Testing for antibodies against Porcine Reproductive and Respiratory Syndrome Virus, Classical and African Swine Fever was negative. There was no further bacteriological examination performed as case history, clinical examination, haematology and pathological examination did not indicate a septicaemia in the litter of the sow.

Due to the clinical, pathological and haematological findings, thrombocytopenic purpura was diagnosed. For the future avoidance of the disease, it was necessary to prohibit further matings of the same boar and sow. The sire of the litter was unknown, as all three boars had contact with the sow and matings were not recorded. Therefore, a genetic study was performed to find the boar, which had fathered the litter. EDTA blood was taken from two piglets and bristles with their roots were collected of the sow and all three boars.

Genomic DNA was extracted from EDTA stabilized blood samples using the Maxwell instrument (Promega). Genomic DNA samples from three piglets, one sow and three boars were genotyped with the using the porcine GeneSeek GGP Porcine BeadChip containing 50,915 single nucleotide variants SNVs. PLINK v1.9 [18] was applied for quality control by removing: SNVs with a call rate below 99%, SNVs with a minor allele frequency below 1% and genotypings of individuals with a call rate below 90%. In total, all six individual genotypes consisting of 41′890 SNVs remained for further analyses. Additionally, PLINK was used for computation of identity by descent (Table 2) and calculation of Mendelian errors for the suspected trios within the six genotyped pigs (Table 3) in order to analyse the family relationship.

**Table 2:** Kinship between individual animals

IID1	IID2	Proportion of identity by descent	Kinship
**Piglet 1**	**Piglet 2**	0.44	Siblings
**Piglet 1**	**Sow**	0.50	Child - mother
**Piglet 1**	**Boar 1**	0.50	Child – father
**Piglet 1**	**Boar 2**	0.00	Unrelated
**Piglet 1**	**Boar 3**	0.00	Unrelated
**Piglet 2**	**Sow**	0.51	Child – mother
**Piglet 2**	**Boar 1**	0.50	Child – father
**Piglet 2**	**Boar 2**	0.00	Unrelated
**Piglet 2**	**Boar 3**	0.00	Unrelated
**Sow**	**Boar 1**	0.00	Unrelated
**Sow**	**Boar 2**	0.00	Unrelated
**Sow**	**Boar 3**	0.03	Unrelated
**Boar 1**	**Boar 2**	0.00	Unrelated
**Boar 1**	**Boar 3**	0.00	Unrelated
**Boar 2**	**Boar 3**	0.16	Slightly related

The higher the proportion of identity by descent (IBD), the more related are two animals. Identical animals would have an IBD of 1. Close relationships, such as siblings or parent – offspring kinships have a proportion of IBD of about 0.5. As expected, the two piglets and the piglets and the mother show a high portion of IBD. The piglets and boar 1 equally show a high proportion of IBD while they are clearly unrelated to the other two boars (pairings in bold letters)

**Table 3:** Number of Mendelian errors with each boar as a proposed father of the nuclear family

Boar	Number of Mendelian errors
**Boar 1**	60
**Boar 2**	15,758
**Boar 3**	15,075

Boar 1 has a much lower number of Mendelian errors than the other two boars, which leads to the assumption that this boar was the sire of the piglets

Both methods revealed the same boar as the sire of the litter with the affecting piglets (Tables 2 and 3). This boar was the oldest of the three boars and had been on the farm for approximately 2 years. The identified boar was subsequently removed from the farm. However, three and a half months later another litter of a different sow showed haemorrhages in the skin. It was therefore important to implement further measures. The farmer was instructed to record all matings in the future. The purchase of a sow planner was recommended, because the matings can be easily recorded. Some programs can evaluate the risk matings and therefore prevent specific genetic diseases [19]. In addition, the documentation on the stock can generally be improved and problems can be detected in advance. The boars should be exchanged regularly to reduce risk of repeated matings. However, as the farm is rather small, implemented measures could not always be applied. Some sows refused matings with a given boar and therefore the boar would be exchanged between groups of sows for insemination. As a further measure, all new boars were of Duroc breed to avoid matings between animals of Landrace and Large White breed. The reduction of risk factors was successful and as of today, no new cases have been reported on this farm.

## Discussion

This report describes the case of a severely affected litter of piglets with thrombocytopenic purpura. The most striking findings were severe haemorrhages in skin and several organs as described in literature [1–3, 7]. However, clinical presentation differed slightly, as the bleedings in the skin were distributed diffusely over nearly the whole skin, but as seen in other cases, more prominent on the ventral abdomen. The bone marrow was in a hyperplastic. Most cases describe a depression of bone marrow [3], while in some cases, especially recovering animals, an increase in myeloid activity is described [4]. The disease has been described to have a biphasic thrombocytopenia: After an initial drop in the amount of thrombocytes in the first few days after birth, thrombocyte numbers may rise again, only to drop once more at around 14 days of age [3, 4]. The tested piglet showed a massive thrombocytopenia, but may have been in the phase of initial improvement or may have been on the path to recovery, which would explain the increased myeloid activity. However, as the piglet was clinically apathetic and moribund, recovery seemed impossible. The massive electrolyte imbalances were attributed to the piglets’ weakness, which prevented them to suckle, and kidney failure following the severe bleedings.

The responsible boar had been at the farm for 2 years, which was the longest period out of the three boars present. The risk of repeated matings of one sow with this boar was highest because of continuous use of the boar. It is remarkable that no cases had been observed before. There are no studies on prevalence of thrombocytopenic purpura, but it seems to be a rare event, which may account for its absence before. It is also possible that mild cases had occurred in the past but were not identified as such. Only one of three affected litters was genetically analysed. It may be that the same boar sired all three litters. As the sows were all from the same breeder, it can be hypothesized, that the affected litters had related mothers. This may explain why more than one sow produced antibodies against the thrombocytes from the same boar.

The attending veterinarian of the farm had never seen the disease before and this might well be the case for most veterinarians in Europe. Most case reports date from a time before the widespread use of artificial insemination, when natural matings with boars on the farm were more common [1–3, 7]. Nowadays, fresh, liquid boar semen is mostly used for artificial insemination [20]. The limited storage time and broad range of available boar semen reduces the probability of repeated matings. The risk of isoimmunisation of the sow against the antibodies of the boar is thus reduced. However, the rising wealth of the Western society [21], where consumers wish for better animal welfare and higher ecological standards, gave rise to special food brands promoting these standards. For example the Swiss organisation for organic food production (Bio Suisse) promotes natural matings, which allows the sow and boar to express natural behaviour [13]. As the number of boars on a farm is limited for economic reasons, it is only natural that the probability of risk matings and number of litters with thrombocytopenic purpura may rise again. With the knowledge of the disease and its pathogenesis as well as with the number of tools at our disposal, it should be possible to avoid losses. Artificial insemination greatly reduces the risk but cases may still occur [10]. Therefore, it is essential to record matings and boars used for artificial insemination to avoid the same pairing in later inseminations of the sow. Recording is best integrated in the normal sow planner software. On farms with natural matings, several boars should be on site and replaced regularly. In case of doubts, there are DNA-based low-cost genotyping tools like SNVs arrays available. These tests were developed to describe population differences, can be used for important applications in livestock, and became available for pigs during the last decade. This allows breed assignment of individual animals, authentication of mono-breed products, as well as parentage verification among several other applications [22].

If there is an affected litter despite all risk management, a case investigation should be conducted. Further risk factors may be identified and avoided in the future. The most important thing is to identify the father and, in cases where piglets are moved after birth [10], the mother of an affected litter. We show in this case report, that this can easily be accomplished by genetic testing. Even in cases, where matings were recorded it may be of value to perform a genetic testing, as recordings may be inaccurate. Genetic testing has become more easily available and affordable. Technics applied in genetics could be implemented for routine use in future, however, practitioners should be made aware of its many advantages.

## Data Availability

Genotyping data are available from the corresponding author on request.

## Abbreviations

IBD: Identity by descent
SNV: Single nucleotide variant
